# Let's Push Things Forward: A Survey on Robot Pushing

**DOI:** 10.3389/frobt.2020.00008

**Published:** 2020-02-06

**Authors:** Jochen Stüber, Claudio Zito, Rustam Stolkin

**Affiliations:** ^1^IRLab, School of Computer Science, University of Birmingham, Birmingham, United Kingdom; ^2^Extreme Robotics Lab (ERL), University of Birmingham, Birmingham, United Kingdom

**Keywords:** robotics, pushing, manipulation, forward models, motion prediction

## Abstract

As robots make their way out of factories into human environments, outer space, and beyond, they require the skill to manipulate their environment in multifarious, unforeseeable circumstances. With this regard, pushing is an essential motion primitive that dramatically extends a robot's manipulation repertoire. In this work, we review the robotic pushing literature. While focusing on work concerned with predicting the motion of pushed objects, we also cover relevant applications of pushing for planning and control. Beginning with analytical approaches, under which we also subsume physics engines, we then proceed to discuss work on learning models from data. In doing so, we dedicate a separate section to deep learning approaches which have seen a recent upsurge in the literature. Concluding remarks and further research perspectives are given at the end of the paper.

## 1. Introduction

We argue that pushing is an essential motion primitive in a robot's manipulative repertoire. Consider, for instance, a household robot reaching for a bottle of milk located in the back of the fridge. Instead of picking up every yogurt, egg carton, or jam jar obstructing the path to create space, the robot can use gentle pushes to create a corridor to its lactic target. Moving larger obstacles out of the way is even more important to mobile robots in environments as extreme as abandoned mines (Ferguson et al., 2004), the moon (King, 2016), or for rescue missions, such as for the Fukushima Daiichi Nuclear Power Plant. In order to save cost, space, or reduce payload, mobile robots are often not equipped with grippers, meaning that prehensile manipulation is not an option. Even in the presence of grippers, objects may be too large or too heavy to grasp.

In addition to the considered scenarios, pushing has numerous beneficial applications that come to mind less easily. For instance, pushing is effective at manipulating objects under uncertainty (Brost, 1988; Dogar and Srinivasa, 2010), and for pre-grasp manipulation, allowing robots to bring objects into configurations where they can be easily grasped (King et al., 2013). Dexterous pushing skills are also widely applied and applauded in robot soccer (Emery and Balch, 2001).

Humans perform skilful manipulation tasks from an early age, and are able to transfer behaviors learned on one object to objects of novel sizes, shapes, and physical properties. For robots, achieving those goals is challenging. This complexity arises from the fact that frictional forces are usually unknown but play a significant role for pushing (Zhou et al., 2016). Furthermore, the dynamics of pushing are highly non-linear, with literal tipping points, and sensitive to initial conditions (Yu et al., 2016). The large body of work on robotic pushing has produced many accurate models for predicting the outcome of a push, some analytical, and some data-driven. However, models that generalize to novel objects are scarce (Kopicki et al., 2017; Stüber et al., 2018), highlighting the demanding nature of the problem.

In this paper, we review the robotic pushing literature. We focus on work concerned with making predictions of the motion of pushed objects, but we also cover relevant applications of pushing for planning and control. This work is primarily targeted at newcomers to robotic pushing, such as Ph.D. students, interested in understanding the evolution of the field. While the main body of this paper focuses on a qualitative analysis of the presented methods, the mathematical treatment is delivered as a set of mini-lectures in the figures. We use the figures to “draw” on the blackboard, thus providing a geometrical intuition for important formalizations used across the literature. Each figure is accompanied by a caption explaining the mathematical content in an accessible yet careful way.

Related to our work is the survey conducted by Ruggiero et al. (2018) which covers the literature on planning and control for non-prehensile dynamic manipulation. Pushing is one of the motion primitives which they consider, among throwing, catching, and others.

In the next section, we provide the problem statement of this survey (section 2). Subsequently, we present the existing literature, beginning with analytical approaches (section 3), under which we also subsume physics engines. We then proceed to discuss data-driven approaches (section 4), including deep learning methods which have recently become very popular in the literature. Finally, we conclude by summarizing the presented approaches and by discussing open problems and promising directions for future research (section 5).

## 2. Problem Statement

**Figure 1:**
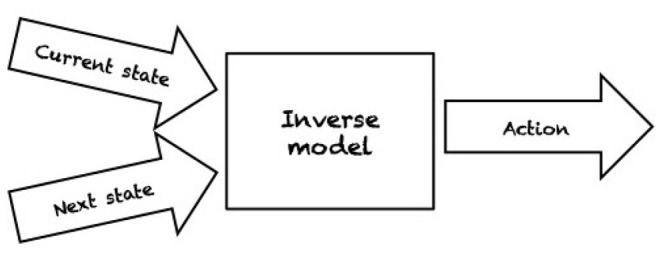
An inverse model computes an action which will affect the environment such that the next desired state (or configuration) is achieved from the current state.

**Figure 2:**
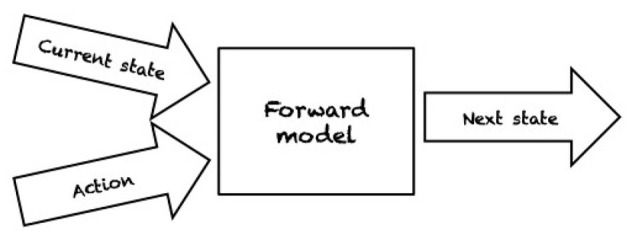
A forward model makes a prediction on how an action will affect the current state of the environment by returning the configuration after the action is taken.

Even in ideal conditions, such as *structured environments* where an agent has a complete model of the environment and perfect sensing abilities, the problems of robotic grasping and manipulation are not trivial. By a complete model of the environment we mean that physical and geometric properties of the world are exactly known, e.g., pose, shape, and friction parameters, as well as the mass of the object we wish to manipulate. In fact, the object to be manipulated is indirectly controlled by contacts with a robot manipulator (e.g., pushing by a contacting finger part). For planning and control, robots need either an *inverse model* (IM) or a *forward model* (FM). IMs compute the action that transforms the current state into the target state (see Figure 1). In contrast, FMs predict the next state resulting from applying an action in the current state (see Figure 2). Depending on the type of model used, a variety of planning and control strategies exist. For instance, an agent may use an FM to imagine the likely outcomes from all possible actions and then choose the action which achieves the most desirable end state (e.g., Zito et al., 2012). An example of an IM-based controller is the work of Igarashi et al. (2010) where a dipole-like vector field is used to compute the direction of motion of a robot pusher such that the object is pushed along a specified path. As manipulation and grasping problems are defined in continuous state and action spaces, finding an optimal continuous control input to achieve the desired state is often computationally intractable.

Even more challenging is the problem of grasping and manipulation in *unstructured environments*, where the ideal conditions of structured environments do not exist. There are several reasons why an agent may fail to build a complete description of the state of the environment: sensors are noisy, robots are difficult to calibrate, and actions' outcomes are unreliable due to unmodeled variables (e.g., friction, mass distribution). Uncertainty can be modeled in several ways, but in the case of manipulation there are typically two types of uncertainty:

- *Uncertainty in physical effects*: occurs when the robot acts on external bodies via physical actions (e.g., contact operations). This interaction transforms the current state of the world according to physical laws which are not fully predictable. For example, a pushed object may slide, rotate or topple with complex motions which are extremely difficult to predict, and involve physical parameters which may not be known. We can think of this as uncertainty on future states.
- *Uncertainty in sensory information*: occurs when some of the quantities that define the current state of the world are not directly accessible to the robot. Thus the necessity to develop strategies to allow the robot to complete tasks in partial ignorance by recovering knowledge of its environment. When executing robotic actions in such cases, sensory uncertainty may propagate to the result of the action.

This paper is concerned with the evolution of models to predict object motions and their application in robotics. Table 1 summarizes the literature at a glance. The papers are classified according to the type of approach implemented. We identify the following six classes.

1. **Purely analytical**. This is mostly seminal work drawn from classical mechanics that uses the quasi-static assumption. To be precise, some of these approaches also venture into dynamic analysis, but with many simplifying assumptions (section 3.1.1).
2. **Hybrid**. Works in this class extend analytical approaches with data-driven methods. Whilst the interactions between objects are still represented analytically, some quantities of interest are estimated based on observations, e.g., the coefficients of friction (section 3.1.2).
3. **Dynamic analysis**. These approaches integrate dynamics in the model (section 3.2.1).
4. **Physics engines**. Here we consider work that employs a physics engine as a “black box” to make predictions about the interactions (section 3.2.2).
5. **Data-driven**. Such models learn how to predict physical interaction from examples (sections 4.1 and 4.2).
6. **Deep learning**. As the data-driven approaches, such models learn how to construct an FM from examples. The key insight is that the deep learning approaches are based on feature extraction (section 4.3).

The features highlighted for each approach are as follows.

- The assumptions made by the authors on their approach. We highlight i) the quasi-static assumption in the model, ii) if it is a seminal work on 2D shapes, and iii) if the method required a known model of the object to be manipulated.
- The type of motion analyzed in the paper, such as 1D, planar (2D translation and 1D rotation around the *x*−axis), or full 3D (3D translation and 3D rotation).
- The aim of the paper. We distinguish between predicting the motion of the object, estimating physical parameters, planning pushes, and analysing a push to reach a stable grasp.
- The model. We distinguish between analytical, constructed from data, and by using a physics simulator.

**Table 1:** Summary of the literature a glance.

		**Assumptions**			**Motion**			**Aim**				**Model**		
		**Quasi-static assumption**	**2D object**	**Known object**	**1D**	**planar**	**3D**	**Motion prediction**	**Parameter estimation**	**Path planning**	**Grasping**	**Analytical**	**Data-driven**	**Physics simulator**
**Purely analytical**	Mason (1982)		✓	✓	✓		✓				✓			
	Mason (1986b)	✓	✓	✓		✓		✓				✓		
	Peshkin and Sanderson (1988a,b)	✓	✓	✓			✓		✓				✓	
	Goyal et al. (1991)	✓	✓	✓		✓			✓					
	Alexander and Maddocks (1993)	✓		✓		✓		✓				✓		
	Lee and Cutkosky (1991)	✓	✓	✓		✓		✓				✓		
	Lynch et al. (1992)	✓		✓		✓			✓			✓		
	Howe and Cutkosky (1996)			✓		✓		✓				✓		
	Mason (1990)			✓			✓			✓		✓		
	Mayeda and Wakatsuki (1991)			✓		✓				✓		✓		
	Akella and Mason (1992, 1998)			✓		✓				✓		✓		
	Narasimhan (1994)	✓	✓	✓		✓				✓		✓		
	Lynch and Mason (1996)	✓	✓	✓		✓				✓		✓		
	Agarwal et al. (1997)		✓	✓		✓				✓		✓		
	Nieuwenhuisen et al. (2005)	✓	✓	✓		✓				✓		✓		
	de Berg and Gerrits (2010)		✓	✓		✓				✓		✓		
	Miyazawa et al. (2005)		✓		✓					✓		✓		
	Cappelleri et al. (2006)	✓		✓		✓				✓		✓		
	Dogar and Srinivasa (2011)	✓		✓			✓				✓	✓		
	Cosgun et al. (2011)		✓	✓		✓				✓				
	Lee et al. (2015)		✓	✓		✓				✓				
	King (2016)			✓		✓				✓				✓
**Hybrid**	Lynch (1993)		✓			✓			✓			✓		
	Yoshikawa and Kurisu (1991)			✓		✓			✓			✓		
	Ruiz-Ugalde et al. (2010, 2011)			✓		✓			✓			✓		
	Zhu et al. (2017)			✓			✓			✓				
	Bauza and Rodriguez (2017)	✓		✓		✓				✓		✓		
**Dynamic analysis**	Brost (1992)					✓		✓				✓		
	Jia and Erdmann (1999)			✓		✓		✓				✓		
	Behrens (2013)			✓		✓		✓				✓		
	Chavan-Dafle and Rodriguez (2015)			✓			✓	✓			✓		✓	
	Woodruff and Lynch (2017)		✓	✓		✓				✓		✓		
**Physic engine**	Zito et al. (2012)	✓		✓			✓			✓			✓	
	Scholz et al. (2014)			✓		✓			✓				✓	✓
	Zhu et al. (2017)			✓			✓		✓					✓
**Data driven**	Moldovan et al. (2012)			✓		✓		✓					✓	
	Ridge et al. (2015)						✓	✓					✓	
	Zrimec and Mowforth (1991)			✓			✓	✓					✓	
	Salganicoff et al. (1993)			✓		✓		✓					✓	
	Walker and Salisbury (2008)					✓		✓					✓	
	Lau et al. (2011)					✓		✓					✓	
	Krivic and Piater (2019)	✓				✓				✓			✓	
	Kopicki et al. (2011, 2017)	✓		✓			✓	✓					✓	
	Stüber et al. (2018)	✓					✓	✓	✓				✓	
	Meriçli et al. (2015)	✓				✓		✓					✓	
**Deep learning**	Denil et al. (2016)						✓		✓				✓	
	Chang et al. (2016)					✓			✓				✓	
	Li et al. (2018)	✓				✓		✓	✓				✓	
	Watters et al. (2017)			✓		✓		✓					✓	
	Fragkiadaki et al. (2015)			✓		✓		✓					✓	
	Ehrhardt et al. (2017)			✓		✓		✓					✓	
	Byravan and Fox (2017)			✓			✓	✓					✓	
	Finn et al. (2016)						✓	✓					✓	

## 3. Analytical Approaches

### 3.1. Quasi-Static Planar Pushing

Early work on robotic pushing focused on the problem of quasi-static planar pushing of sliding objects. In a first phase, several researchers, following pioneering work by Matthew T. Mason, approached the problem analytically, explicitly modeling the objects involved and their physical interactions whilst drawing on theories from classical mechanics. More recently, this tradition has moved to extend analytical models with more data-driven methods.

#### 3.1.1. Purely Analytical Approaches

To briefly introduce the problem, *planar pushing* (Mason, 1982), refers to an agent pushing an object such that pushing forces lie in the horizontal support plane while gravity acts along the vertical. Both pusher and pushed object move only in the horizontal plane, effectively reducing the world to 2D. Meanwhile, the *quasi-static assumption* (Mason, 1986b) in this context means that the involved objects' velocities are small enough that inertial forces are negligible. In other words, objects only move when pushed by the robot. Instantaneous motion is then the consequence of the balance between contact forces, frictional forces, and gravity. The quasi-static assumption makes the problem more tractable, yielding simpler models. A key challenge in predicting the motion of a pushed object under manipulation is that the distribution of pressure at the contact between object and supporting surface is generally unknown. Hence, the system of frictional forces that arise at that contact is also indeterminate (Mason, 1982).

Mason (1982, 1986a) started the line of work on pushing, proposing the voting theorem as a fundamental result. It allows one to find the sense of rotation of a pushed object given the pushing direction and the center of friction without requiring knowledge of the pressure distribution. Drawing on this seminal work, Peshkin and Sanderson (1988a,b) found bounds on the rotation rate of the pushed object given a single-point push. Following that, Goyal et al. (1991) introduced the *limit surface* which describes the relationship between the motion of a sliding object and the associated support friction given that the support distribution is completely specified. Under the quasi-static assumption, the limit surface allows one to convert the generalized force applied by a pusher at a contact to the instantaneous generalized velocity of the pushed object. Alexander and Maddocks (1993) considered the case when only the geometric extent of the support area is known, and described techniques to bound the possible motions of the pushed object. While the limit surface provides a powerful tool for determining the motion of a pushed object, there exists no convenient explicit form to construct it. In response to this challenge, Lee and Cutkosky (1991) proposed to approximate the limit surface as an ellipsoid to improve computational time. However, their approximation requires knowledge of the pressure distribution. Marking a milestone of planar pushing research, Lynch et al. (1992) applied the ellipsoidal approximation to derive a closed-form analytical solution for the kinematics of quasi-static single-point pushing, including both sticking and sliding behaviors. Subsequently, Howe and Cutkosky (1996) explored further methods for approximating limit surfaces, including guidance for selecting the appropriate approach based on the pressure distribution, computational cost, and accuracy.

**Figure 3:**
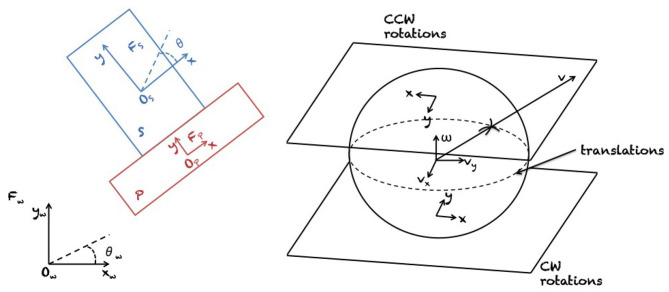
The slider *S* (blue) is a rigid object in the plane ℝ^2^, and its configuration space is ℝ^2^ × Θ, i.e., 2D translation and one rotation over the *x*−axis. The slider is pushed by a rigid pusher *P* (red) at a point or set of points of contact. A world frame *F*_*w*_ with origin *O*_*w*_ is fixed in the plane, and a slider frame *F*_*w*_ with origin *F*_*s*_ is attached to the center of friction of the slider *S*. The configuration ${\left(x_{w},y_{w},\theta_{w}\right)}^{⊺}$ describes the position and orientation of the slider frame *F*_*s*_ relative to the world frame *F*_*w*_. Similarly, a pusher frame *F*_*p*_ with origin *O*_*p*_ and its configuration is computed. On the right side of the figure, the relation between the unit motion vector $\text{v}={\left(v_{x},v_{y},\omega \right)}^{⊺}$ and the center of rotation of frame *F*_*s*_ is described by the projection shown from the unit motion sphere to the tangent planes (one for each rotation sense). The line at the equator of the sphere represents translations. Reproduced from Lynch and Mason (1996).

**Figure 4:**
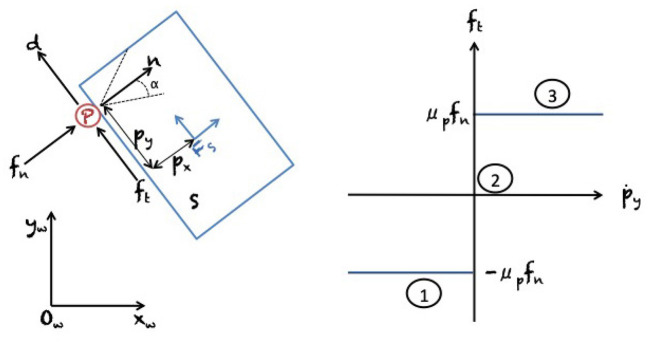
**(Left)** Planar pushing system with world frame *F*_*w*_ (with origin *O*_*w*_) and a slider *S* (blue) with frame *F*_*w*_ as described in Figure 3. The pusher *P* (red) is interacting with the slider on one point of contact. It impresses a normal force *f*_*n*_, a tangential friction force *f*_*t*_, and a torque τ about the center of mass. The normal force *f*_*n*_ is in the direction of the normal vector *n* of the contact point between pusher and slider, and α = arctanμ_*p*_ is the angle of the friction cone assuming μ_*p*_ as the coefficient of friction. The terms *p*_*x*_ and *p*_*y*_ describe, respectively, the normal and the tangential distance between the pusher *P* and the center of friction of the slider *S*. **(Right)** Coulomb's frictional law for the planar pushing system on the left-hand figure. Coulomb's law states that the normal and tangential forces are related by *f*_*t*_ = μ_*p*_*f*_*n*_. Three contact modes are defined. (1) Sliding right in which friction acts as a force constraint; (2) Sticking in which friction acts as a kinematic constraint; and (3) Sliding left in which friction acts as a force constraint. Reproduced from Bauza et al. (2018).

Results on the mechanics of planar pushing have been used for *planning and control* of manipulator pushing operations. To begin with, Mason (1990) showed how to synthesize robot pushing motions to slide a block along a wall, a problem later also studied by Mayeda and Wakatsuki (1991). Akella and Mason (1992, 1998) analyzed the series of pushes needed to bring a convex polygon to a desired configuration. Narasimhan (1994) and Kurisu and Yoshikawa (1995) studied the problem of moving an object among obstacles by pushing with point contact. Lynch and Mason (1996) comprehensively studied stable pushing of a planar object with a fence-shaped finger, considering mechanics, control, and planning. First, they derived conditions for stable edge pushing, considering the case where the object will remain attached to the pusher without slipping or breaking contact. Based on this result, they then used best-first search to find a path to a specified goal location. Figure 3 shows the proposed representation of motions by Lynch and Mason (1996). Agarwal et al. (1997) proposed an algorithm for computing a contact-preserving push plan for a point-sized pusher and a disk-shaped object. Nieuwenhuisen et al. (2005) utilized compliance of manipulated disk-shaped objects against walls to guide their motion. They presented an exact planning algorithm for 2D environments consisting of non-intersecting line segments. Subsequently, de Berg and Gerrits (2010) improved this approach from a computational perspective and presented push-planning methods both for the contact-preserving case and less restrictive scenarios. Miyazawa et al. (2005) used a rapidly-exploring random tree (RRT) (LaValle, 1998) for planning non-prehensile manipulation, including pushing, of a polyhedron with three degrees of freedom (DOF) by a robot with spherical fingers. They do not allow for sliding and rolling of robot fingers on the object surface. Cappelleri et al. (2006) have solved a millimeter scale 2D version of the peg in the hole problem, using Mason's models for quasi-static manipulation and an RRT-based approach for planning a sequence of pushes. Figure 4 presents a graphical representation of planar motions and Coulomb's frictional law that governs such systems (see the figure caption for further details).

More recently, Dogar and Srinivasa (2011) employed the ellipsoidal approximation of the limit surface to plan robust push-grasp actions for dexterous hands and used them for rearrangement tasks in clutter. To use results for planar pushing, they assumed that objects do not topple easily. Furthermore, they assumed that the robot has access to 3D models of the objects involved. Cosgun et al. (2011) presented an algorithm for placing objects on cluttered table surfaces, thereby constructing a sequence of manipulation actions to create space for the object. However, focusing on planning, in their 2D manipulation they simply push objects at their center of mass in the desired direction. Lee et al. (2015) presented a three-stage hierarchical approach to planning sequences of non-prehensile and prehensile actions. First, they find a sequence of qualitative contact states of the moving object with other objects, then a feasible sequence of poses for the object, and lastly a sequence of contact points for the manipulators on the object.

In summary, although of fundamental importance for understanding the mechanisms of pushing, analytical approaches are limited by their own inherent complexity. The assumptions around which they are built do not hold in real applications, e.g., a robot link in contact with an object does not produce a single-point contact or the frictional forces are not constant over a supporting surface. Proofs of concept for demonstrating the validity and stability of such methods are generally confined to carefully chosen testing scenarios or special applications, e.g., the frictionless millimeter scale peg-in-the-hole scenario in Cappelleri et al. (2006). Extensions to non-convex or novel-shaped objects challenge analytical approaches. Yet, controllers and planners can easily be synthesized for specified objects and environments. Due to the deterministic nature of the models, they do not implicitly account for uncertainty in the state description or the predictions. Nonetheless, an analytical method can be employed as a black box to forward-simulate the effect of a given action within a planner. For instance, King (2016) developed a series of push planners for open-loop non-prehensile rearrangement tasks in cluttered environments. Before considering more complex scenarios, they used a simple analytical approach for forward-simulation of randomly sampled time-discrete controls within an RRT-based planner. They tested their planners on two real robotic platforms, the home care robot HERB with a seven DOF arm, and the NASA rover K-Rex.

#### 3.1.2. Complementing Analytical Approaches With Data-Driven Methods (Hybrid)

**Figure 5:**
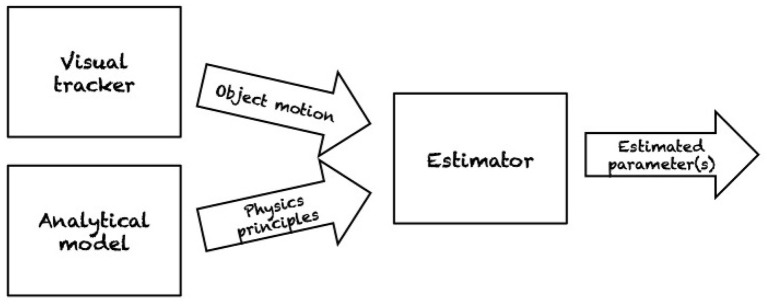
A classical workflow for estimating relevant physical parameters of a pushed object. A robotic pusher performs a set of push operation on an object which is typically tracked using vision. Simpler approach employs markers on the object for more accurate estimations. An analytical model of the motion for the target object is also employed. Sensory data and physical principles are the inputs of the estimator. As output, the estimator provides with an estimate of the desired parameters, e.g., friction distribution or center of mass. In Lynch (1993) the estimated parameters are also used for recognizing objects based on their (estimated) physical properties.

Transitioning to the second phase of planar pushing research, multiple factors have contributed a shift toward more *data-driven approaches*. For one thing, much of the previous work makes minimal assumptions regarding the pressure distribution. While convenient, those methods lead to conservative strategies for planning and control, providing only worst case guarantees. Furthermore, while assumptions regarding the pressure distribution in previous work were often minimal, other strong assumptions were frequently made to derive results analytically. Hence, more recent work has set out to validate common assumptions such as the ubiquitous quasi-static assumption. Additionally, purely analytical models do not take into account the stochastic nature of pushing in the sense that pushes indistinguishable to sensor and actuator resolution have empirically been found to produce variable results (Yu et al., 2016). Instead of making minimal or strong assumptions about parameters, they can instead be *estimated* based on observations. Several researchers have explored this approach to deal with the inherent uncertainties of this problem (section 1). Figure 5 summarizes a classical workflow for estimating relevant physical parameters of a pushed object.

Lynch (1993) presented methods both for estimating the relevant friction parameters by performing experimental pushes, and for recognizing objects based on their friction parameters. Similarly, Yoshikawa and Kurisu (1991) described how a mobile robot with a visual sensor can estimate the friction distribution of an object and the position of the center of friction by pushing and observing the result. Yet, both of these approaches discretise the contact patch into grids so that they are either imprecise if the approximation is too coarse or suffer from the curse of dimensionality when using a fine-grained approximation. Ruiz-Ugalde et al. (2010, 2011) formulated a compact mathematical model of planar pushing. Assuming that the object's base shape is given, their robot explored object-table and finger-object friction coefficient parameters. Zhou et al. (2016) developed a method for modeling planar friction, proposing a framework for representing planar sliding force-motion models using convex polynomials. Notably, they also showed that the ellipsoid approximation is a less accurate special case of this representation. Zhou et al. (2017) extended the convex polynomial model to associate a commanded position-controlled end effector motion to the instantaneous resultant object motion. They modeled the probabilistic nature of object-to-surface friction by sampling parameters from a set of distributions. They presented the motion equations for both single and multiple frictional contacts and validated their results with robotic pushing and grasping experiments on the dataset published by Yu et al. (2016). That dataset comprises planar pushing interactions with more than a million samples of positions of pusher and slider, as well as interaction forces. Push interaction is varied along six dimensions, namely surface material, shape of the pushed object, contact position, pushing direction, pushing speed, and pushing acceleration. Using their dataset, they characterized the variability of friction, and evaluated the most common assumptions and simplifications made by previous models of frictional pushing. They provide an insightful table that lists the assumptions and approximations made in much of the work cited in this section. More recently, Bauza et al. (2019) have published Omnipush, an extensive dataset of planar pushing behavior that extends their previous work. It comprises 250 pushes for each of 250 objects. The pushing velocity is constant and chosen so that the interaction is close to quasi-static. They improved on their previous dataset by providing RGB-D sensor data in addition to tracking data, increasing object diversity, adding controlled variation of the objects mass distribution, and creating benchmarks to evaluate models. Finally, Bauza and Rodriguez (2017) used a data-driven approach to model planar pushing interaction to predict both the most likely outcome of a push and, as a novelty, its expected variability. The learned models, also trained on the dataset by Yu et al. (2016), rely on a variation of Gaussian processes whilst avoiding and evaluating the quasi-static assumption by making the velocity of the pusher an input to the model. However, the learned models are specific to the particular object and material. Transfer learning is left for future work.

### 3.2. Physics Engines and Dynamic Analysis

While the quasi-static assumption may be reasonable in a variety of situations, other problems call for dynamic models of pushing. One popular approach to achieving this is using a physics engine. Before covering this field, we first consider work concerned with dynamic pushing that does not recur to physics engines.

#### 3.2.1. Dynamic Analysis

Using dynamic analysis, Brost (1992) investigated the problem of catching an object by pushing it, i.e., determining the pushing motions that lead to a pusher-object equilibrium. This work was motivated by dealing with uncertainty in positioning, generating plans that work also in the worst case. Jia and Erdmann (1999) investigated dynamic pushing assuming frictionless interaction between pusher and object. Behrens (2013) instead studied dynamic pushing but assumed infinite friction between pusher and object. Chavan-Dafle and Rodriguez (2015) considered planning non-prehensile in-hand manipulation with patch contacts. They described the quasi-dynamic motion of an object held by a set of frictional contacts when subject to forces exerted by the environment. Given a grasp configuration, gripping forces, and the location and motion of a pusher, they estimate both the instantaneous motion of the object and the minimum force required to push the object into the grasp. To this end, complex contact geometries are broken up into rigid networks of point contacts. More recently, Woodruff and Lynch (2017) extended earlier work by Lynch and Mason (1999) on dynamic manipulation primitives, including pushing, by proposing a method for motion planning and feedback control of hybrid, dynamic, and non-prehensile manipulation tasks.

#### 3.2.2. Physics Engines

A large body of work related to pushing makes use of *physics engines*. Commonly used examples of such engines include Bullet Physics, the Dynamic Animation and Robotics Toolkit (DART), MuJoCo, the Open Dynamics Engine (ODE), NVIDIA PhysX, and Havok (Erez et al., 2015). Those engines allow for 3D simulation but 2D physics engines exist, as well, e.g., Box2D. While some physics engines have been designed for graphics and animation, others have been developed specifically for robotics. In the first category, visually-plausible simulations are key while physically-accurate simulations are essential for many robotics applications. Most physics engines today use impulse-based velocity-stepping methods to simulate contact dynamics. As this requires solving NP-hard problems at each simulation step, more tractable convex approximations have been developed, highlighting the trade-off between computational complexity and accuracy present in those engines (Erez et al., 2015). 3D physics engines use a Cartesian representation where each body has six DOF and joints are modeled as equality constraints in the joint configuration space of the bodies. In robotics, where joint constraints are ubiquitous, using generalized coordinates is computationally less expensive and prevents joint constraints from being violated.

For a *comparison* of physics engines, we refer the reader to two recent studies (Erez et al., 2015; Chung and Pollard, 2016). Erez et al. (2015) compared ODE, Bullet, PhysX, Havok, and MuJoCo. It should be noted that the study was written by the developers of MuJoCo. They introduced quantitative measures of simulation performance and focused their evaluation on challenges common in robotics. They concluded that each engine performs best on the type of system it was tailored to. For robotics, this is MuJoCo while gaming engines shine in gaming-related trials, whereby no engine emerges as a clear winner. Chung and Pollard (2016) compared Bullet, DART, MuJoCo, and ODE with regard to contact simulations whilst focusing on the predictability of behavior. Their main result is that the surveyed engines are sensitive to small changes in initial conditions, emphasizing that parameter tuning is important. Another evaluation of MuJoCo was carried out by Kolbert et al. (2017) who evaluated the contact model of MuJoCo with regard to predicting the motions and forces involved in three in-hand robotic manipulation primitives, among them pushing. In the course, they also evaluated the contact model proposed by Chavan-Dafle and Rodriguez (2015). They found that both models make useful yet not highly accurate predictions. Concerning MuJoCo, they state that its soft constraints increase efficiency but limit accuracy, especially in the cases of rigid contacts and transitions in sticking and slipping at contacts.

**Figure 6:**
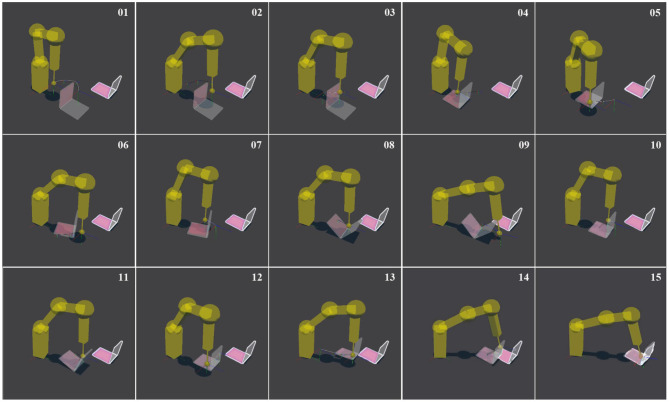
Simulation of a Katana robot arm equipped with a spherical finger that plans a sequence of pushes to move an L-shaped object, called polyflap (Sloman, 2006) to a goal state. The plan is created by using a physics engine (PhysX) to predict the outcome of a push operation. Image 01 shows the initial pose. The wire-framed L-shaped polyflap is a “phantom” to indicate the desired goal state. The goal pose is translated from the initial pose by 28 cm and rotated by 90°. Image 02 shows the collision-free trajectory to bring the end effector to the start pose of the first push. Images 01–04 show the first push which makes the polyflap tip over. Images 05–09 show a series of pushes which culminate in the polyflap resting in an unstable equilibrium pose along its folded edge. Images 12 and 13 show a sideways push. Images 14 and 15 show the final frontal push which aligns the polyflap with the target configuration. Courtesy of Zito et al. (2012).

Researchers have applied physics engines in multifarious ways to study robotic pushing. To begin with, physics engines have been used in RRT-based planners to forward-simulate pushes. Zito et al. (2012) presented a two-level planner that combines a global RRT planner operating in the configuration space of the object, and a local planner that generates sequences of actions in the robot's joint space that will move the object between a pair of nodes in the RRT. In this work, the experimental set-up consists of a simulated model of a tabletop robot manipulator with a single rigid spherical fingertip which it uses to push a polyflap (Sloman, 2006) to a goal state. To achieve this, the randomized local planner utilizes a physics engine (PhysX) to predict the object's pose after a pushing action. Erroneous estimates and uncertainty in the motion is not directly taken into account by the planner. Hence, a re-planning stage is required when the actual motion differs from the prediction by more than a user-defined threshold. Figure 6 shows a sequence of actions planned by the two-level planner for pushing a polyflap to a desired configuration (see caption for further details). Similarly, King (2016) incorporated a dynamic physics engine (Box2D) into an RRT-based planner to model dynamic motions such as a ball rolling. To reduce planning complexity, they considered only dynamic actions that lead to statically stable states, i.e., all considered objects need to come to rest before the next action. Another application of physics engines in robotic pushing was proposed by Scholz et al. (2014). In what they refer to as Physics-Based Reinforcement Learning, an agent uses a physics engine as a model representation. Hence, a physics engine can be seen as a hypothesis space for rigid-body dynamics. They introduced uncertainty using distributions over the engine's physical parameters and obtained transitions by taking the expectation of the simulator's output over those random variables. Finally, Zhu et al. (2017) utilized a physics engine for motion prediction, learning the physical parameters through black-box Bayesian optimization. First, a robot performs random pushing actions on an object in a tabletop set-up. Based on those observations, the Bayesian learning algorithm tries to identify the model parameters that maximize the similarity between the simulated and observed outcomes. To support working with different objects, a pre-trained object detector is used that maps observed objects to a library of 3D meshes and estimates the objects' poses on that basis. Once the physical parameters have been identified, they are used to simulate the results of new actions.

Finally, while physics engines and dynamic analysis offer great value for robotic applications, e.g., by taking into consideration dynamic interaction and 3D objects, they nevertheless require explicit object modeling and extensive parameter tuning. Another approach, which we consider next, is to learn how to predict object motions from data.

## 4. Learning to Predict From Examples

This part of the literature is based on learning predictive models for robotic pushing from data. We first review work on qualitative models and then consider models that make metrically precise predictions. In both of those sections, we do not include work that uses deep learning techniques. We dedicate a separate section to such approaches, given the current research interest in that area and the large number of papers being published.

### 4.1. Qualitative Models

Much work on qualitative models revolves around the concept of *affordances*. The term affordance was invented by Gibson (1979) and generally refers to an action possibility that an object or environment provides to an organism. Although it has originated from psychology, the concept has been influential in various domains, among them robotics. Sahin et al. (2007) discussed affordances from a theoretical perspective while laying emphasis on their use in autonomous robotics. Min et al. (2016) provided a recent survey of affordance research in developmental robotics.

Although the concept of affordances is typically associated with learning “high-level” actions from contexts, e.g., pushing an object in a clutter scene when grasping is not available, in this paper we focus on investigations that extend affordances to the effects of an action too. Ugur et al. (2011) considered an anthropomorphic robot that learns object affordances as well as effect categories through self-interaction and self-observation. After learning an FM as a mapping between object affordances and effects, the proposed method can make plans to achieve desired goals, emulate end states of demonstrated actions, and cope with uncertainty in the physical effects by monitoring the plan execution and taking corrective actions using the perceptual structures employed or discovered during learning. While much previous work has focused on affordance models for individual objects, Moldovan et al. (2012) learned affordance models for configurations of multiple interacting objects with push, tap, and grasp actions for achieving desired displacements or rotations, as well as contacts between objects, for selecting the appropriate object and action for the subsequent step. Their model is capable of generalizing over objects and dealing with uncertainty in the physical effects. Ridge et al. (2015) developed a self-supervised online learning framework based on vector quantization for acquiring models of effect classes and their associations with object features. Specifically, they considered robots pushing household objects and observing them with a camera. Limitations of such learning approaches are that they do not tend to generalize well to novel objects and actions. This is also due to a lack of interpretation and understanding of novel contexts. In fact, self-interaction and self-observation are mainly limited by the ability of the robot to discover novel scenarios and learning opportunities by itself (see also section 5.1).

**Figure 7:**
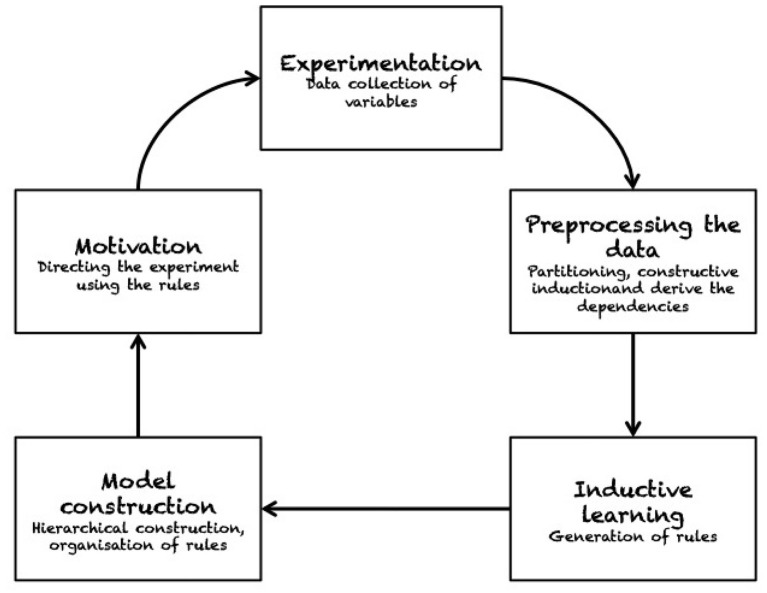
The sequence of operations adopted by Zrimec and Mowforth (1991) to construct their causality learning model. The robot learns by interacting with the environment in an unsupervised fashion. The system can autonomously discover knowledge, as e.g., whether an action generates a push on an object. The “motivation” module guarantees that the system is driven toward acquiring more knowledge about the robot/environment interaction. Reproduced from Zrimec and Mowforth (1991).

Considering *other qualitative approaches* than those related to affordances, Zrimec and Mowforth (1991) developed an algorithm for knowledge extraction and representation to predict the effects of pushing. In their experiment, a robot performs random pushes and uses unsupervised learning on those observations. Their method involves partitioning, constructive induction and determination of dependencies (see Figure 7). Hermans et al. (2013) developed a method for predicting contact locations for pushing based on the global and local object shape. In exploratory trials, a robot pushes different objects, recording the objects' local and global shape features at the pushing contacts. For each observed trajectory, the robot computes a push-stability or rotate-push score and maps shape features to those scores by means of regression. Based on that mapping, the robot can search objects of novel shape for features associated with effective pushes. Experimental results are reported for a mobile manipulator robot pushing household objects in a tabletop set-up.

While learned affordances, and other qualitative models, can be useful in various scenarios, other applications require the ability to predict the effects of pushing more precisely, e.g., by explicitly predicting six DOF rigid body motions. We consider efforts made to achieve precise predictions in the next section.

### 4.2. Metrically Precise Models

Early seminal work by Salganicoff et al. (1993) presented a vision-based unsupervised learning method for robot manipulation. A robot pushes an object at a rotational point contact and learns an FM of the action effects in image space. Subsequently, they used the FM for stochastic action selection in manipulation planning and control. The scenarios considered in this work are relatively simple in that the pusher remains within the friction cone of the object and the contact only has one rotational DOF. Yet, this work takes an approach that is markedly different from analytical models discussed before. Instead of estimating parameters such as frictional coefficients explicitly, the authors *encode* that information *implicitly* in the mapping between actions and their effects in image space. Similarly, Walker and Salisbury (2008) learned a mapping between pushes and object motion as an alternative to explicitly modeling support friction. Set in a 2D tabletop environment, a robot with a single finger pushes objects and uses an online, memory-based local regression model to learn manipulation maps. To achieve this, they explicitly detect the object's shape using a proximity sensor and fit a shape to the thus obtained point cloud. A method for handling objects with more complex shapes was proposed by Lau et al. (2011). In their work, a robot, while being of simple circular shape itself, aims to deliver irregularly shaped flat objects to a goal position by pushing them. The objects that they consider are chosen to exhibit quasi-static properties. Collecting several hundred random example pushes as training data, an FM is learned using non-parametric regression, similar to the approach taken by Walker and Salisbury (2008). Also tackling the problem of object delivery, Krivic and Piater (2019) proposed a modular method for pushing objects in cluttered and dynamic environments that can work with unknown objects without prior experience. Drawing on their previous work (Krivic et al., 2016; Krivic and Piater, 2018), the authors' approach comprises a space-reasoning module, a strategy module, and an adaptive pushing controller which learns local IMs of robot-object interaction online. While their approach shows a high success rate of object delivery for objects with quasi-static properties in simulated and real-world environments, it does not take into account object orientation and depends on vision-based measurements of the object motion.

**Figure 8:**
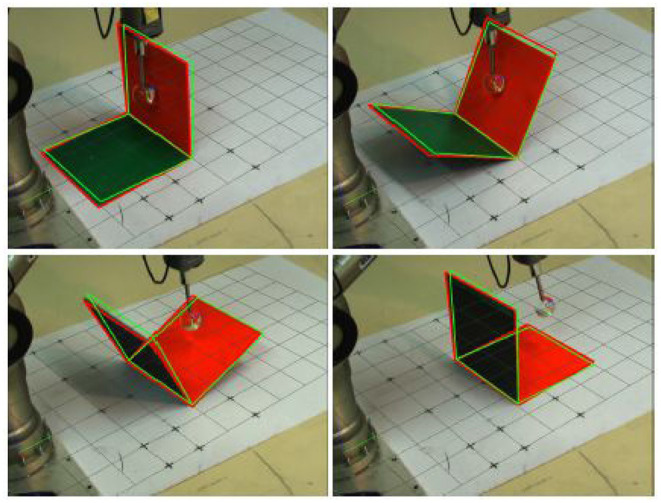
The example shows the interaction between a 5-axis Katana robotic manipulator and an L-shape object, called polyflap (Sloman, 2006). A set of contact experts are learned as probability densities for encoding geometric relations between parts of objects under a push operation. This approach allows these experts to learn from demonstration physical properties, such as non-penetration between an object and a table top, without explicitly representing physics knowledge in the model. The green wire frame denotes the prediction whilst the red wire frame denotes the visual tracking. Courtesy of Kopicki et al. (2011).

**Figure 9:**
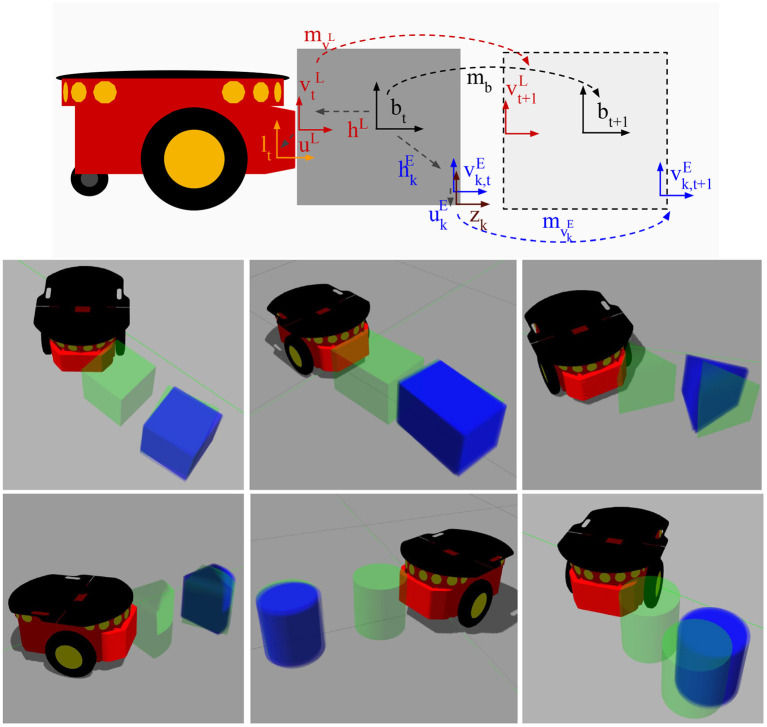
**(Top)** graphical representation of the feature-based predictors for push operations. The global motion of the object after a push is described by the rigid body transformation *m*_*b*_. This transformation is unknown to the robot. However, the robot can estimate it by learning a set of local predictors for the motions $m_{\upsilon^{L}}$ and $m_{\upsilon_{k}^{E}}$, for *k* = 1, …, *N*_*E*_. The rigid body transformations *h*^*L*^ and $h_{k}^{E}$ describe the estimated contacts on the object's surface w.r.t. the estimated global frame of the object, *b*. Since the object is assumed to be rigid, this relation does not change over time, thus once the local motions $m_{\upsilon^{L}}$ and $m_{\upsilon_{k}^{E}}$ are estimated, *b*_*t*+1_ can be estimated by using the relations *h*^*L*^ and $h_{k}^{E}$. **(Bottom)** resulting predictions. initial object pose (green, in contact with robot), true final object pose (green, displaced), and predictions (blue). Courtesy of Stüber et al. (2018).

Kopicki et al. (2011) presented two data-driven probabilistic methods for predicting 3D motion of rigid bodies interacting under the quasi-static assumption. First, they formulated the problem as regression and subsequently as density estimation. Figure 8 shows an example of the interaction between a 5-axis Katana robotic manipulator and a polyflap (Sloman, 2006). In Kopicki et al. (2017) they extended this work further. Their architecture is modular in that multiple object- and context-specific FMs are learned which represent different constraints on the object's motion. A product of experts is used which, contrary to mixture models, does not add but multiply different densities. Hence, all constraints, e.g., those imposed by the robot-object contact and multiple object-environment contacts, need to be satisfied so that a resulting object motion is considered probable. This formulation facilitates the transfer of learned motion models to objects of novel shape and to novel actions. In experiments with a robot arm, the method is compared with and found to outperform the physics engine PhysX tuned on the same data. For learning and prediction, their algorithms require access to a point cloud of the object. A further extension of this approach is presented in Stüber et al. (2018). In this work, the authors aim to contribute to endowing robots with versatile non-prehensile manipulation skills. To that end, an efficient data-driven approach to transfer learning for robotic push manipulation is proposed. This approach combines and extends two separate strings of research, one directly concerning pushing manipulation (Kopicki et al., 2017), and one originating from grasping research (Kopicki et al., 2016). The key idea is to learn motion models for robotic pushing that encode knowledge specific to a given type of contact, see the work by Kopicki et al. (2016) for further details. Figure 9 presents a graphical representation of the feature-based predictors as well as resulting predictions across object shapes. In an previously unseen situation, when the robot needs to push a novel object, the system first establishes how to create a contact with the object's surface. Such a contact is selected among the learned models, e.g., a flat contact with a cube side or a contact with a cylindrical surface. At the generated contact, the system then applies the appropriate motion model for prediction, similarly to that of Kopicki et al. (2017). The underlying rationale for this approach to prediction is that predicting on familiar ground reduces the motion models' sample complexity while using local contact information for prediction increases their transferability (Krivic et al., 2016).

Meriçli et al. (2015) similarly presented a case-based approach to push-manipulation prediction and planning. Based on experience from self-exploration or demonstration, a robot learns multiple discrete probabilistic FMs for pushing complex 3D objects on caster wheels with a mobile base in cluttered environments. Subsequently, the case models are used for synthesizing a controller and planning pushes to navigate an object to a goal state whilst potentially pushing movable obstacles out of the way. In the process, the robot continues to observe the results of its actions and feeds that data back into the case models, allowing them to improve and adapt.

Metrically precise models have become a stable trend of research in the field of robotic push manipulation. Their probabilistic nature elegantly deals with state and motion uncertainties. Nonetheless, real applications of robot pushing may require higher levels of reasoning to be useful assistants. For example, a warehouse robot may need to fill shelves with many boxes via push operations. This would also require planning multiple sequences of actions where some earlier placements may lead to a sub-optimal final configuration. Efficient planning solutions would need to reason about gathering critical information concerning the task space or propagating the uncertainty in the action's effects to future states. We present some suggestions on how to deal with these types of problems in the final remarks (see sections 5.2 and 5.3).

### 4.3. Deep Learning Approaches

*Deep learning* commonly refers to methods that employ artificial neural networks to learn models from data. It has been used in robotic pushing to estimate physical parameters, predict the outcome of pushing actions, and for planning and control. Previously, we have seen work concerned with estimating physical parameters of the environment from data. Deep learning has been used to address the same problem. Denil et al. (2016) studied the learning of physical properties such as mass and cohesion of objects in a simulated environment. Using deep reinforcement learning, their robots learn different strategies that balance the cost of gathering information against the cost of inaccurate estimation.

Instead of explicitly estimating physical parameters, another approach is learning a *dynamics model*. Several studies have investigated learning general physical models or “physical intuition” directly from image data. Chang et al. (2016) presented the Neural Physics Engine, a deep learning framework for learning simple physics simulators. They factorize the environment into object-based representations and decompose dynamics into pairwise interaction between objects. However, their evaluation is limited to simple rigid body dynamics in 2D. Li et al. (2018) proposed Push-net, a deep recurrent neural network to tackle the problem of quasi-static planar pushing to re-orient and re-position objects. Their approach requires only visual camera images as input and remembers pushing interactions using a long short-term memory (LSTM) module. An auxiliary objective function estimates the COM of the object, thus encoding physics into their model. They trained their model in simulation and tested it on various objects in simulation and on two real robots, with results indicating that Push-Net is capable of generalizing to novel objects.

**Figure 10:**
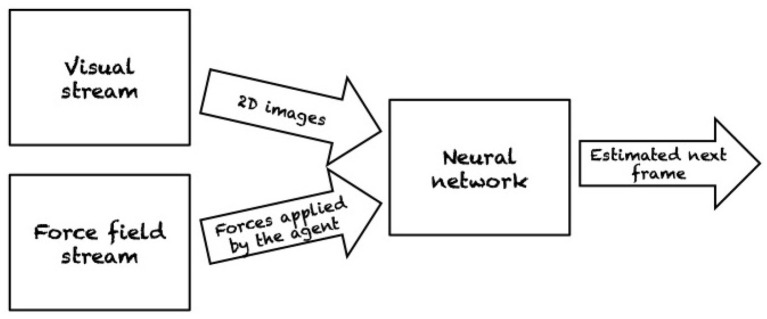
Frame-centric model for motion prediction of billiard balls. The model takes as input the 2D image of the billiard and the forces applied by the agent to make predictions about the future configurations of the balls. Reproduced from Fragkiadaki et al. (2015).

**Figure 11:**
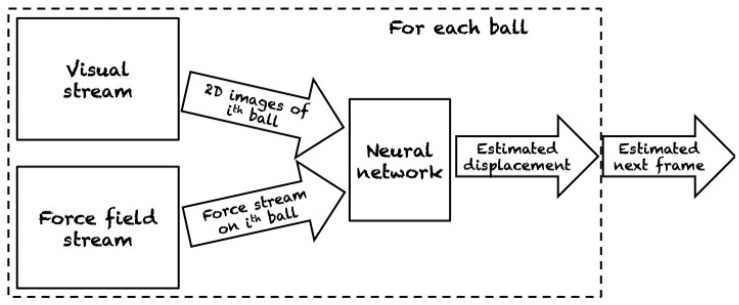
Object-centric model for motion prediction of billiard balls. The system predicts the future configurations of the balls by individually modeling the temporal evolution of each ball. In this scenario, predicting the velocities of each ball is sufficient for computing the next configuration of the billiard. Reproduced from Fragkiadaki et al. (2015).

Watters et al. (2017) introduced the Visual Interaction Network, a model for learning the dynamics of a physical system from raw visual observations. First, a convolutional neural network (CNN) generates a factored object representation from visual input. Then, a dynamics predictor based on interaction networks computes predicted trajectories of arbitrary length. They report accurate predictions of trajectories for several hundred time steps using only six input video frames. Yet, their experiments are also limited to rather simple environments, namely 2D simulations of colored objects on natural-image backgrounds. Similarly, Fragkiadaki et al. (2015) also used an object-centric formulation based on raw visual input for dynamics prediction. Based on object-centric visual glimpses (snippets of an image), the system predicts future states by individually modeling the behavior of each object. A graphical representation of this model is presented in Figure 10. After training in different environments by means of random interaction, they also use their model for planning actions in novel environments, in this case moving balls on a 2D table (Figure 11). Ehrhardt et al. (2017) constructed a neural network for end-to-end prediction of mechanical phenomena. Their architecture consists of three components: a CNN extracts features from images which are updated by a propagation module, and decoded by an estimation module. What their network outputs is a distribution over outcomes, thus explicitly modeling the inherent uncertainty in manipulation prediction. In terms of experiments, they study the relatively simple problem of a small object sliding down an inclined plane.

Moving toward more complex scenarios, Byravan and Fox (2017) introduced SE3-NETS, a deep neural network architecture for predicting 3D rigid body motions. Instead of RGB images, their network takes depth images as input, together with continuous action vectors, and associations between points in subsequent images. SE3-NETS segment point clouds into object parts and predict their motion in the form of *SE*(3) transformations. They report that their method outperforms flow-based networks on simulated depth data of a tabletop manipulation scenario. Furthermore, they demonstrate that it performs well on real depth images of a Baxter robot pushing objects. However, their approach requires that associations between depth points are provided. They aim to learn those automatically in future work and to apply SE3-NETS to non-rigid body motion, recurrent prediction, and control tasks. A different approach to learning dynamics from images was taken by Agrawal et al. (2016). They jointly learn FMs and IMs of dynamics of robotic arm operation that can be used for poking objects. In doing so, they extract features from raw images and make predictions in that feature space. In real-world experiments with Baxter, their model is used to move objects to target locations by poking. In order to cope with the real world, their model requires training on large amounts of data. By poking different objects for over 400 h, their robot observed more than 100, 000 actions.

Most of the studies presented this section make use of *object-centric representations* to model dynamics. Other approaches predict motion without such representations. For instance, Finn et al. (2016) developed an action-conditioned video prediction system which predicts a distribution over pixel motions only based on previous frames. No information concerning object appearance is provided to the model. It borrows that information from previous frames and merges it with model predictions. It is this mechanism that allows the model to generalize to previously unseen objects. By conditioning predictions on an action, the model can effectively imagine the action's consequences. As with previously presented deep learning models, this approach also requires large amounts of data to perform well in real-world situations. Hence, the authors have collected a dataset of 59, 000 robot pushing motions (frames associated with the action being applied) on different objects. While their results demonstrate that no object-centric representation is required for prediction, the authors argue that such representations are a promising direction for research as they provide concise state representations for use in reinforcement learning.

We have seen how artificial neural networks can be used to model the dynamics of physical systems. In addition to that, deep reinforcement learning has been used to learn control policies in the field of robotic pushing. Many of those approaches make use of dynamics models so that they can be seen as complementary to the work presented before. We do not provide a detailed review of this very active field here and refer the reader to Levine et al. (2015), Levine et al. (2016), Finn and Levine (2017), and Ghadirzadeh et al. (2017) for overviews of such work.

## 5. Final Remarks

In this paper we have provided an overview of the problem of robot pushing and summarized the development of the state-of-the-art, focusing on the problem of motion prediction of the object to be pushed. We have also covered some aspects of relevant applications of pushing for planning and control.

Typical approaches have been classified as (i) purely analytical, (ii) hybrid, (iii) dynamic analysis, (iv) physics engines based, (v) data-driven, and (vi) deep learning. Representative work for each of these categories has been listed for readers to have a general overview of the field and its state-of-the-art from the earlier work in the 1980s to the most recent approaches.

A set of assumptions in the proposed methods have been highlighted. Earlier work has mostly investigated motion prediction with the quasi-static assumption to get rid of complex dynamics and provided the groundwork to understand the mechanics for pushing 2D shapes. This seminal work has been extended to more realistic scenarios involving 3D objects to be pushed. Nonetheless, as we have seen there are two types of uncertainty that affect manipulation problems: (i) prediction uncertainty and (ii) state uncertainty. Unfortunately, purely analytical approaches are computationally tractable only under the assumption that the geometrical properties of the object to be pushed are known *a priori* and the dynamics are negligible (e.g., Mason, 1990; Mayeda and Wakatsuki, 1991). Key physical properties that would affect the prediction, e.g., mass distribution or friction coefficients, were typically assumed to be known or possible to estimate on the fly, as in Yoshikawa and Kurisu (1991), by combining data-driven methods with the analytical mechanics of pushing.

More recently, a few efforts were made toward robot pushers that can also deal with state uncertainty. By relaxing the assumption that the model of the object to be pushed is known, the robot typically perceives the object as a point cloud or RGB image to estimate the geometric properties, such as pose and shape, before even attempting a motion prediction, see Fragkiadaki et al. (2015) and Stüber et al. (2018).

Two strands of approaches can be identified: data-driven and deep learning techniques. They are similar in that they both define (or extract) some informative features as a basis for learning and model predictions in a probabilistic framework to estimate an action's most likely outcome given the information available, e.g., an image of the scene or contact models.

Qualitative models have made use of the concept of affordances for learning a mapping between object features and candidate actions, which they then employ for planning. For manipulation tasks, however, the planner also needs to learn the relationship between actions and their effects by creating a mapping from actions to observable end states and their variability. End states can be represented as displacements after a push operation or a set of contacts for a prehensile operation. This enables us to synthesize controllers with multiple classes of actions and their expected effects which are then employed for planning. Affordances can be learned for 3D motions of a single or multiple interacting objects, but they do not generalize well to novel objects or actions. Erroneous predictions can be dealt with at execution time by triggering a re-planning procedure. In contrast to qualitative models, metrically precise models are concerned with directly learning a mapping between observable features (e.g., contacts or geometrical features) and their effects in the context of manipulation. Factorizing robot-object and object-environments contacts as (probabilistic) experts enables a robot pusher to generalize predictions across object categories, e.g., demonstrated by Kopicki et al. (2017). Physical properties such as impenetrability can also be learned implicitly by the experts and transferred to novel contexts. The quasi-static assumption limits the model to predictions of object motion in the next time-step, but roll-out predictions can be executed to approximate continuous operations. A second, more recent strand is the application of deep learning techniques to learning a physical intuition of the mechanics of pushing from visual data, see Fragkiadaki et al. (2015). Automatic feature extraction can be used to estimate uncertain state information such as the COM from raw data (e.g., RGB images), as well as to make predictions on interaction dynamics. Controllers for specific robots can also be learned directly by the models, but there is a lack of evidence on whether these controllers could be transferred across robot platforms, or how their performance would degrade when dealing with novel objects or actions. Nevertheless, the main disadvantage of these approaches is the amount of data required. While Stüber et al. (2018) and Meriçli et al. (2015) can learn from as little as a few hundred pushes, Push-net and the other deep learning approaches require hundreds of times more data.

While some typical problems still require a better solution, new challenges and requirements are emerging in the field. To make pushing an essential motor primitive in practical robotics, the challenges are either currently under investigation in research group worldwide or need to be investigated in the future. Following we list some suggested trends of open problems that we have identified.

### 5.1. Understanding and Semantic Representation

The scene is typically perceived as an RGB image or a point cloud. However, for robot pushing, we need to be able to identify pushable objects from static ones. Labeling can be done but it is very expensive in terms of human labor. Converting from source image data to geometrical shapes, and from geometrical shape to semantic representation will be beneficial for the robot. Once the robot can identify probable dynamic objects within a semantic map of the environment it would be able to interact with the environment prioritizing those objects and improving its understanding.

### 5.2. Sensory Fusion and Feedback

Multiple sensor inputs are nowadays available for robotic system. Instead of solely relying on vision, other sources of information should be used to close the loop of the manipulation. Tactile, proprioception, and visual feedback should be fused together to enable the robot to perform complex manipulation and recover from failures. Generative models, such recurrent neural networks, can learn manipulative operations from multiple sensory sources. This enables the robot to compensate for missing or corrupted input data, as well as to predict the next sensory state and the associated expected error with respect to the next observed sensory state, which can then be used for implementing adaptive behaviors. For readers interested in sensory fusion for manipulative tasks, we refer to the work of Yang et al. (2016) as an interesting starting point.

### 5.3. Explicitly Modeling Uncertainty in the Model

Due to a lack of perfect perception abilities, it is not unusual that robots have to operate with an incomplete description of their environment. In robot pushing, but more generally in the problem of manipulation, the robot needs to generate a set of contacts to interact with other objects. When the pose of the object to be manipulated is unknown, what is the best way to create a robust set of contacts? In the case of planning for dexterous manipulation, our previous work in Zito et al. (2013) has demonstrated that approaching directions that maximize the likelihood of gathering (tactile) information are more likely to achieve a successful set of contacts for a grasp. This was tested in the case when, due to imperfect perception abilities, the pose of the object to be grasped remained uncertain. This empirically suggests that reasoning about the uncertainty leads to more robust reach-to-grasp trajectories with respect to object-pose uncertainty. Similarly, selecting an action for physical effects (e.g., pushing, push, and grasp) should benefit from incorporating state uncertainty with respect to the initial pose estimate of the object. Finally, we highlight the complexity of incorporating uncertainty in models for pushing which results from the multi-modal stochasticity inherent to the task demonstrated by Yu et al. (2016).

### 5.4. Cooperative Robots and Multiple Contacts Pushing

Moving large-scale objects is a common problem in warehouses that can be achieved with cooperative robots. Besides the problem of sharing information between them and coordinating the efforts, a new challenge arises from the manipulation point of view. Multiple contacts pushing is hard to predict, especially when the actions are carried by multiple agents. Scholars interested in the problem of multiple contacts pushing are referred to the works of Lynch (1992) and Erdmann (1998) as interesting starting points.

### 5.5. Real-World Applications

Although the theory behind motion prediction is well-established and applications to simple, structured scenarios have been made, the combination of the existing methods with any industrial applications has not yet been achieved. Robots in warehouses can navigate freely and deliver goods, however, no robotic system is capable of exploiting pushing operations for novel items in novel situations, such as inserting a box of varied produce onto an over-the-head store shelf. Theoretical solutions are rarely reliable in practical engineering applications, hence many sophisticated practical approaches will be needed in the future. Very recently, the robotics community has realized that one of the main issues that prevent a methodological and stable advancement in the field is the lack of standardized benchmarks and metrics for objective evaluations of different approaches. Following the example of fields such as computer vision and natural language process, large and diverse datasets are required to provide the equivalent richness for physical understanding and its application to robot manipulation tasks. A recent attempt in this direction is presented in Bauza et al. (2019).

## Author Contributions

JS was the main author of this paper and collected the literature. CZ was the leading supervisor of this work and he has co-written the paper. RS has co-supervised and funded this project.

### Conflict of Interest

The authors declare that the research was conducted in the absence of any commercial or financial relationships that could be construed as a potential conflict of interest.
